# Silicon increases the phosphorus availability of Arctic soils

**DOI:** 10.1038/s41598-018-37104-6

**Published:** 2019-01-24

**Authors:** Jörg Schaller, Samuel Faucherre, Hanna Joss, Martin Obst, Mathias Goeckede, Britta Planer-Friedrich, Stefan Peiffer, Benjamin Gilfedder, Bo Elberling

**Affiliations:** 1 Environmental Geochemistry, Bayreuth Center for Ecology and Environmental Research (BayCEER), University Bayreuth, 95440 Bayreuth, Germany; 2 Center for Permafrost (CENPERM), University of Copenhagen, DK-1350 Copenhagen, Denmark; 3 Experimental Biogeochemistry, BayCEER, University Bayreuth, Dr.-Hans-Frisch-Str. 1-3, 95448 Bayreuth, Germany; 4 Max Planck Institute for Biogeochemistry, Hans-Knöll-Straße 10, 07745 Jena, Germany; 5 Department of Hydrology, BayCEER, University of Bayreuth, D-95440 Bayreuth, Germany; 6 Limnological Research Station, Bayreuth Center for Ecology and Environmental Research (BayCEER), University Bayreuth, 95440 Bayreuth, Germany

## Abstract

Phosphorus availability in soils is an important parameter influencing primary production in terrestrial ecosystems. Phosphorus limitation exists in many soils since a high proportion of soil phosphorus is stored in unavailable forms for plants, such as bound to iron minerals or stabilized organic matter. This is in spite of soils having a high amount of total soil phosphorus. The feasibility of silicon to mobilize phosphorus from strong binding sites of iron minerals has been shown for marine sediments but is less well studied in soils. Here we tested the effect of silicon on phosphorus mobilization for 143 Artic soils (representing contrasting soil characteristics), which have not been affected by agriculture or other anthropogenic management practices. In agreement with marine studies, silicon availabilities were significantly positive correlated to phosphorus mobilization in these soils. Laboratory experiments confirmed that silicon addition significantly increases phosphorus mobilization, by mobilizing Fe(II)-P phases from mineral surfaces. Silicon addition increased also soil respiration in phosphorus deficient soils. We conclude that silicon is a key component regulating mobilization of phosphorous in Arctic soils, suggesting that this may also be important for sustainable management of phosphorus availability in soils in general.

## Introduction

Phosphorus (P) is a key element for metabolic pathways and carbon (C) turnover on Earth. All organisms need P for their primary functioning. Some examples include energy turnover and cellular integrity (phospholipids) or genetic information (~9% of DNA and RNA are P atoms) as discussed by Westheimer^1^. However, bioavailable P is often scarce in ecosystems^2^. Accordingly, P is one of the elements mostly limiting primary production and yield of crop plants^3^, which has led to massive amounts of P fertilisation in agriculture at the global scale^2^. Due to the strong demand of agricultural systems for P, the sustainability of mineable P for fertilizer production is a matter of debate^2^.

A main influence on the content and availability of P in soils is the biogeochemical conditions in terms of mineral composition^4^. The P content of soils is not necessarily low, however a high proportion of this P is stored in plant unavailable forms such as organic P^5^, or is bound/adsorbed as inorganic P to e.g. aluminum (Al), iron (Fe)oxides, or calcium (Ca) minerals, depending on soil pH^6^, soil diagenesis stage^7^ and mineral composition. At soil pH >6.5 inorganic P is predominately immobilised as calcium phosphate minerals, whereas at lower pH values P tends to be bound/adsorbed by soluble Fe, manganese (Mn), Al, or their hydrous oxides^8^. At neutral pH inorganic P reacts with silicate minerals (adsorption to weathered silicates like clay minerals)^9^. Hence, the distribution of inorganic P between Ca, Fe, Al or Si fractions is highly dependent on soil pH in combination with the mineral composition depending on parent material and soil diagenesis stage.

The P binding to soil minerals is lowest when it is associated with silicate minerals (e.g. clay minerals with high silicon availability)^8,9^. The Si fractions in soils are composed of dissolved Si (free in soil solution or adsorbed to Fe or Al oxides/hydroxides), amorphous forms (e.g. the biogenic phytoliths or the minerogenic silica nodules), poorly crystalline forms (e.g. secondary quartz), and crystalline forms (the primary silicates like mica, feldspars or quartz and the secondary silicates e.g. clay minerals)^10^. Silicon concentration in soil solution in terrestrial ecosystems varies over at least two orders of magnitude in soils and sediments (0.01 to 2.0 mM L^−1^)^11^, and is mainly controlled by vegetation type, parent material, and soil diagenesis stage^12,13^. When analysing soils from different climate regions, different parent material and different vegetation forms, Saccone, *et al*.^14^ found a large range of Si availability from 1.8 to 58 mg g^−1^, using alkaline extraction by Na_2_CO_3_. However, Si availability in terrestrial soils (especially those used by agriculture) is potentially declining due to effects of ecosystem management^15–17^ and the yearly withdrawal by crop harvest, since many crop plants are Si accumulators^11^.

For marine ecosystems, Si is known to interfere with Fe mineralogy competing with P for binding sites, increasing P mobilization and availability^18,19^. These marine studies clearly showed the importance of Si for P availability for different time periods in Earth’s history. Another element that is important for marine P turnover is Ca, which binds P at high pH by co-precipitation of less soluble Ca-phosphates together with Ca-carbonates. This is initiated at a pH of ~7^20,21^ at elevated Ca availability in soils. Calcium availability in soils is commonly in the range of 0 to ~40 mg g^−1^ (Mehlich-3 extractable)^22^.

There is however little information about Si availability in terrestrial soil systems in regard to interactions with P availability. It has been suggested that in terrestrial systems Si fertilization is able to increase the P content of plants by potentially increasing P availability^23,24^. However, except of a few sorption experiments using pure minerals^25–27^ and a few studies about the Si effects on P mobilization in soils^28–31^, less is known so far on how Si is interfering with P mobilization in soils. Another important link of Si and Ca in marine ecosystems is an intricate coupling with the C cycle. Silicon availability increases C fixation by diatoms, while Ca increases C fixation by coccolithophores^32^. For grass dominated environments recent studies have shown a relation to plant lignin synthesis^33^ and a positive correlation between organic matter Si content and organic matter decomposition rates^34,35^, suggesting an interdependence. It was also recently shown that increasing Si availability mobilizes P and organic matter from binding to peat and accelerates formation of CO_2_ and CH_4_ in peat porewaters^36^. However, the underlying mechanisms are not well understood. For Ca a negative effect on soil respiration is known due to a reduction in P availability (at least at high soil pH) and by flocculation of organic matter through Ca^2+^ cation bridges which stabilizes the organic matter^37,38^.

The aim of this study is to determine the importance of soil Si in competition with Ca availability for P mobilization, analyzing the results in the contexts of potential effects on soil C turnover. We collected 143 soil samples from four Arctic locations representing contrasting Arctic environments in terms of climate, landscape history, vegetation and expected soil organic C content/age. These soils were chosen as they were not affected by agriculture or other anthropogenic management practices, and exhibit considerable variation in soil types and soil properties across the Arctic^39^. Additionally, we chose two of the Greenlandic soils, which differed in P, Si and Ca availability as well in pH. These samples were used for further laboratory incubation experiments where the Si and Ca availability of the soils were modified without changing soil pH. Our hypothesis was that Si is positively related to P mobilization, mobilizing inorganic P from formerly unavailable pools (strongly bound to mineral surfaces), whereas Ca decreases P mobilization at high soil pH. In addition we expected positive effects of Si (via P mobilization and organic C mobilization) and negative effects of Ca (via P mobilization and organic C stabilization) on soil respiration.

## Results

### Silicon and calcium affect soil phosphorus mobilization

**Figure 1:**
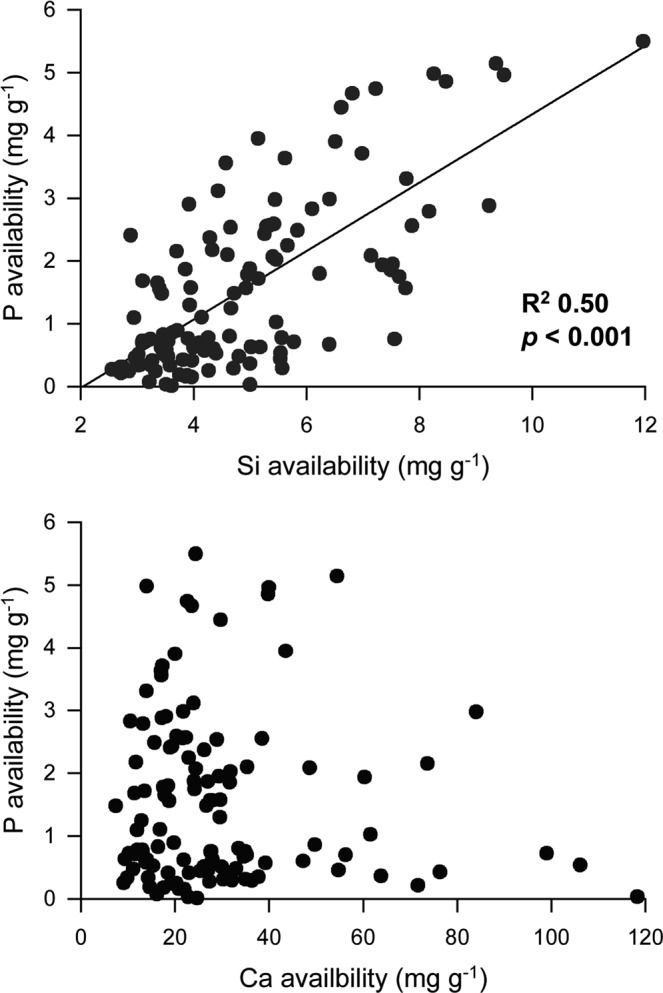
Soil phosphorus (Mehlich-3 extraction) availability in relation to silicon (alkaline extraction) and calcium(Mehlich-3 extraction) availability. Data from 143 soil samples of mineral soils (permafrost and mineral active layer) from the transects (Lena Delta, Abisko and Svalbard) and from three sites from Greenland (Peary Land, Zackenberg and Disko Island).

**Figure 2:**
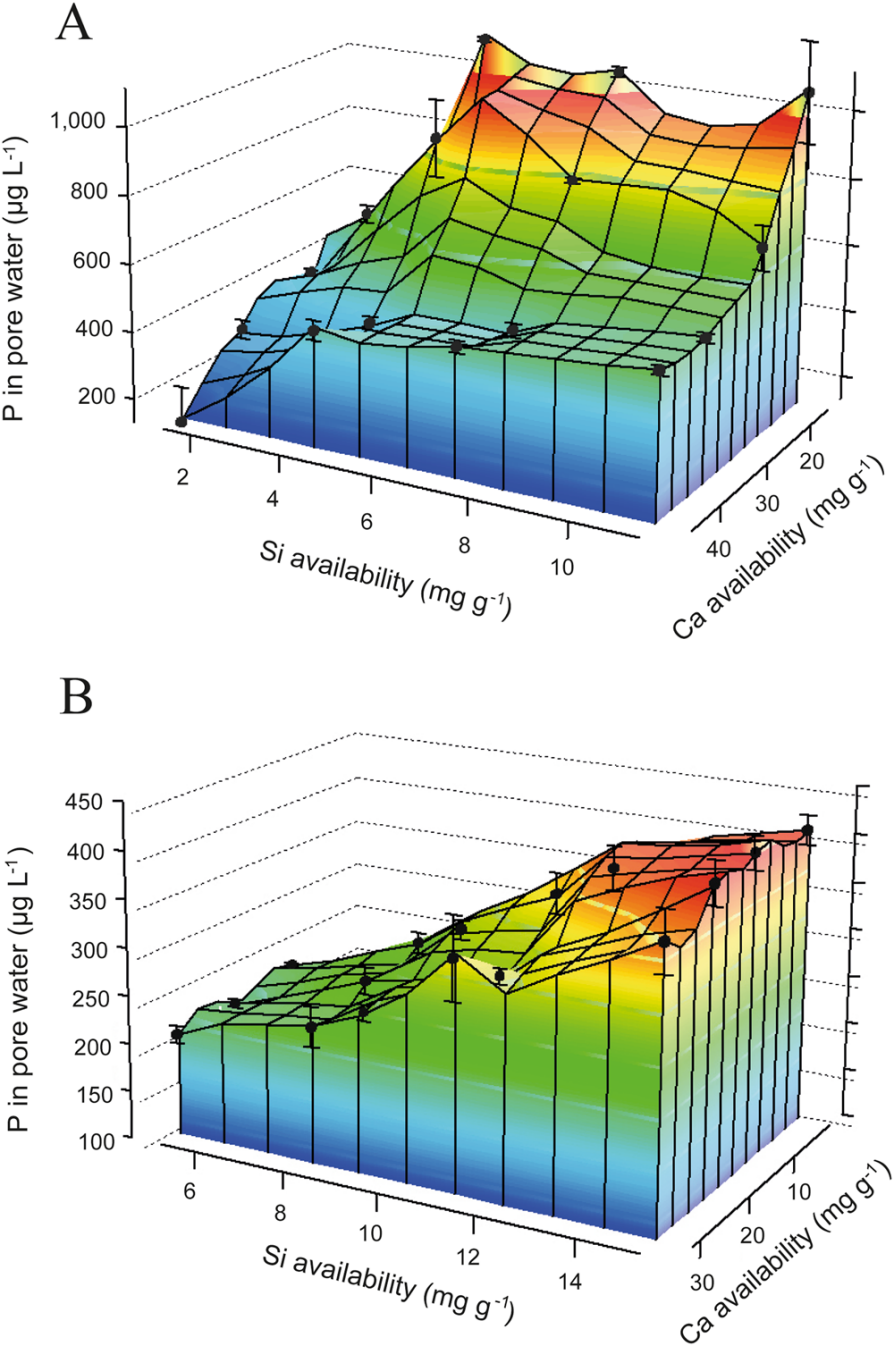
Phosphorus concentrations in pore waters with increasing Si and Ca additions. Phosphorus concentrations in porewaters for two different soils (Peary Land (**A**) with pH 8.4 and Disko (**B**) with pH of 5.6) related to Si and Ca availabilities after 3 months of incubation (note that Si and Ca availability units are in mg g^−1^ soil). Values are from blue (low concentration) to red (high concentration).

For about 150 soils Arctic soils, covering contrasting soil types and soil properties from different landscapes units across the Arctic, we found a substantial correlation (R^2^ = 0.5, *p* < 0.001) between P and Si availability, but no significance between P and Ca availability (Fig. 1). The laboratory experiments revealed a significant increase in P mobilization for both soils upon addition of Si (Fig. 2, Table S1), while the addition of Ca resulted in decreasing P mobilization for high soil pH values in Peary Land (Fig. 2a). The observation of Ca availability decreasing P mobilization appears to be pH controlled, as the negative relationship was only found for Peary Land soil with its high pH values.

### Silicon and calcium controls on phosphorus binding

**Figure 3:**
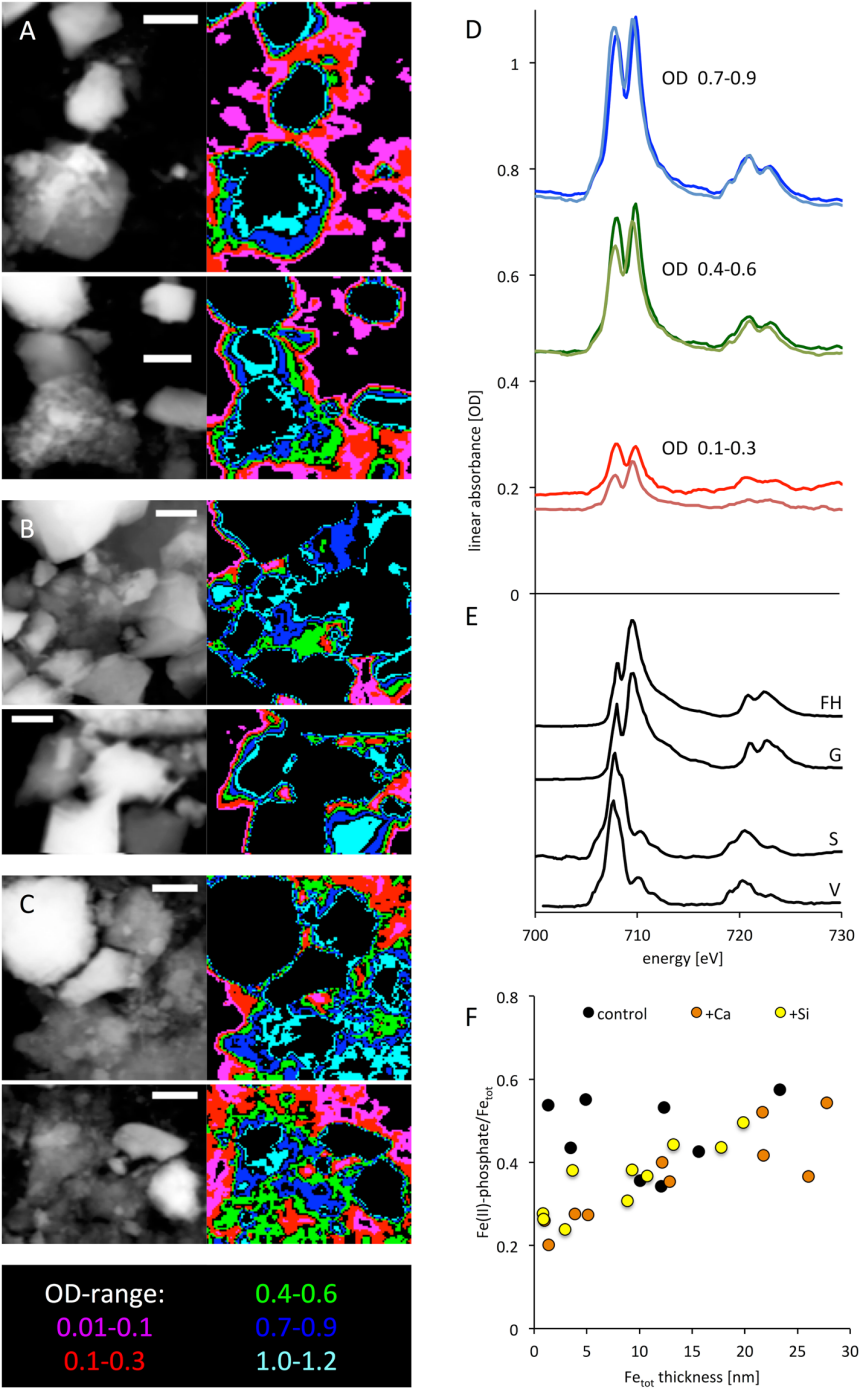
Soil P binding affected by Si and Ca measured by XAS. Soft X-ray NEXAFS study on Fe-speciation at the surface of soil particles. Linear absorbance images and respective thickness masks based on the optical density (OD), that were used to extract spectra of two samples of the Peary Land soils each of the soil after Si addition (**A**), the original soil (**B**), and the soil after Ca addition (**C**), at the end of the three months incubation experiments. Scale bars 2 µm. (**D**) Exemplary Fe 2p spectra of the original soil (dark colours) and after Si addition (bright colours) for three thickness ranges. (**E**) spectra of pure reference compounds ferrihydrite (FH), goethite (G), siderite (S), and vivianite (V), that were used for the linear decomposition of the measured spectra. (**F**) Fe(II)-phosphate/Fe_tot_ ratios plotted against the respective cumulative thickness of Fe_tot_, based on the quantitative fits of all acquired datasets.

To unravel the underlying mechanisms of Si and Ca affecting P mobilization, we analyzed the Fe phases in detail, as they tend to be the major binding sites for phosphate in soils and may be influence by the presence of Si and Ca. Quantitative NEXAFS spectra were extracted from the spatially resolved Fe2p spectromicroscopy datasets of soil particles (Peary Land, end of incubation experiment) and averaged over specific thickness ranges of the particles for the soil after Si addition (Fig. 3A), the original soil (Fig. 3B), and the soil after Ca addition (Fig. 3C). Figure 3D shows one dataset for each thickness range of the original soil (dark colours) and after highest Si addition (bright colours). In thin regions (especially average ODs between 0.1 and 0.3), we observed a strong decrease in both the overall spectral signature and in the dominant peak at ~707.7 eV which is specific for Fe(II) (Fig. 3D). The spectral signature was fitted best using the reference spectrum of an Fe(II)-phosphate phase (here vivianite, Fig. 3E) in contrast to an Fe(II)-carbonate phase. In contrast, for thick regions (average ODs between 0.7 and 0.9), this decrease in the Fe(II)-phase was not significant (Fig. 3D). Hence, we found a decrease in an Fe(II)-phosphate phase after Si addition, indicating a mobilization of P from the mineral phase. This is likely by Si competition for sorption sites at the surface of soil particles. Figure 3F finally shows the quantitative results of all fits (i.e. two datasets of each original soil, after Si and after Ca addition, with five increasing cumulative thickness ranges each, i.e. showing a gradient from surface-dominated towards bulk-dominated spectra). Here, the addition of both Si and Ca resulted in the reduction of an Fe(II)-phase that was interpreted as Fe(II)-phosphate, again restricted to the outermost surface (~5 nm cumulative thickness) of the soil particles(Fig. 3F). In summary, NEXAFS measurements indicated a mobilization of P from mineral surface-bound Fe(II)-phosphate phases by Si and Ca.

### Interdependencies of silicon, calcium and phosphorus potentially affecting soil respiration

**Figure 4:**
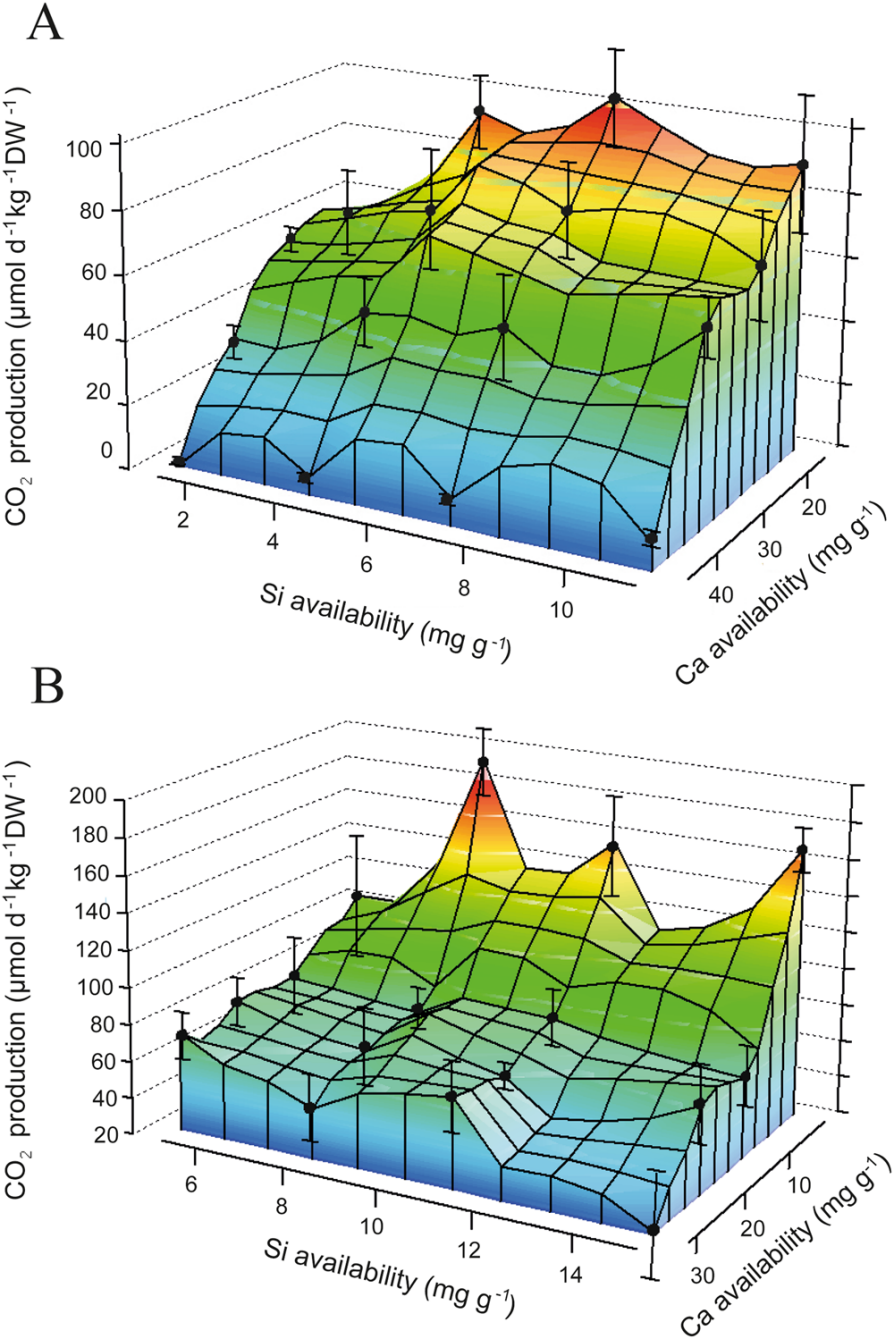
CO_2_ release affected by Si and Ca. Soil CO_2_ production for two different soils (Peary Land (**A**) with pH 8.4 and Disko (**B**) with pH of 5.6) related to Si and Ca availabilities after 3 months of incubation (note that Si and Ca availability units are in mg g^−1^ soil). Values are from blue (low concentration) to red (high concentration).

Our results indicate that the pronounced effect of Si and Ca on P mobilization and availability is also related to soil respiration (Fig. 4). Thus respiration in the soils studied here is potentially P limited, and an increase in P availability is promoting microbial respiration of soil organic matter. The soil respiration was significantly positively related to Si availability for Peary Land and Disko (Fig. 4, Table S1). A significant interaction was also seen between Si and Ca availability for Peary Land and Disko, and Ca availability alone had a negative effect on soil respiration for Peary Land and for Disko (Fig. 4). For Peary Land (low original Si availability) the Si fertilization had a stronger effect on CO_2_ release compared to Disko (higher original Si availability).

To demonstrate that the positive Si effect on respiration is caused by the Si-induced increase of P availability, we conducted an additional treatment where P as NaH_2_PO_4_ was added in concentrations equivalent to the amount mobilized by the highest Si treatment. The results showed a comparable (no significant difference) increase in CO_2_ release for the highest Si addition treatment and the P addition treatment (as NaH_2_PO_4_) for both soils, Peary Land (Si addition: 85 ± 23 µmol d^−1^ kg^−1^ DW^−1^ and P addition treatment 87 ± 10 µmol d^−1^ kg^−1^ DW^−1^) and Disko (Si addition: 154 ± 12 µmol d^−1^ kg^−1^ DW^−1^ and P addition treatment 172 ± 21 µmol d^−1^ kg^−1^ DW^−1^).

## Discussion

The pronounced correlation of Si and P availability for the 143 soil samples from the Arctic (Fig. 1) and the strong mobilization of P from soils by Si addition (Fig. 2) is supporting our hypothesis that Si is positively related to P availability and is important for mobilizing P from previously unavailable phases. This could be explained by a decrease in surface-bound Fe-P phase under elevated Si availability (Fig. 3), as shown previously for Fe(III) minerals^25^. Such processes have also been demonstrated by Schwertmann and Fechter^40^ and Sigg and Stumm^41^ and form the chemical mechanism for explaining the Si effect on P availability for marine systems. Schwertmann and Fechter^40^ as well as Kingston *et al*.^42^ showed that Si (as silicic acid) interferes with the surface charge of Fe minerals. Sigg and Stumm^41^ found that Si (as silicic acid) is deprotonated at the surface of Fe minerals due to the surface charge of the Fe minerals. This reaction changes the Fe mineralogy to colloidal iron silicates or iron hydroxyl silicates (in the region close to the mineral surface by Si adsorption). The same studies clearly showed that Si (as silicic acid) is strongly competing with P (as H_2_PO_4_^−^) for binding sites at the Fe minerals, with slightly lower binding affinity of silicic acid compared to P. However, soil pore water concentrations of silicic acid in natural soils/sediments can be very high^13^, potentially shifting the competition between silicic acid and P towards Si. The effect of Si modifying Fe mineralogy to at least Si coated Fe minerals has been confirmed by several studies (e.g.^26,27,43,44^). The interaction of Si with the surface of the Fe minerals explains the occurrence of the strongest Si effects on Fe-P minerals in thin regions at the end of the three months incubation experiment (reduction of Fe-P phases by Si directly at the mineral surface during soil respiration experiment) (Fig. 3). Silicon as a primary factor interfering with P mobility and binding is well known for marine systems but mostly neglected in soil science, despite being potentially important due to the strong plant demand for P and declining mineable P resources^2^. The positive Si effect on P mobilization may be not restricted to Fe minerals as Koski-Vähälä, *et al*.^45^ suggested that Si may also be able to decrease P binding to Al-oxides, thereby increasing P availability. At soil pH levels in the range of those from Peary Land (pH 8.4), precipitation of carbonates and co-precipitation of calcium phosphate may occur, as shown by^46^, supporting our hypothesis of Ca decreasing P mobilization. In the case of Ca mobilizing P from Fe minerals (Fig. 3), we assume that free P was subsequently bound to Ca and precipitates as calcium phosphate minerals resulting in a decrease in P mobility, as shown by^47^.

The possitive effects of Si on soil respiration (Fig. 4) confim our hypothesis that Si is positively related to repiration, and may be explained in two ways. First (primary effect), Si increases P availability, which is promotes soil respiration in P-deficient systems^48,49^. Secondly, as a minor effect, Si has also been shown to desorb organic C from mineral binding sites (e.g. goethite) as suggested above for P^50^. Phosphorus with its high binding affinity to soil minerals mobilizes organic C and thus potentially increasing C mineralization, as shown previously for amorphous Al hydroxide (am-Al(OH)_3_)^51^. Such a competition of P and C for mineral sorption sites was also shown for goethite^52^. Silicon, with strong bonding affinity to soil minerals comparable with C and P, may mobilize both elements, and may also affect the respiration of soil organic matter directly. The negative effect of Ca on soil respiration confirms our hypothesis and could be explained by reducing P availability (at least at soil pH >8)^6^ and by flocculation of organic matter through Ca^2+^ cation bridges which stabilizes the organic matter^37,38^. That may also explain why we find no interdependency between Ca and P in soils from the highly different soil types and soil properties from different landscapes^39^.

Our data on the interdependency of Si and P availability in soils are based on highly different soil types and soil properties from different landscapes^39^. These results are consistent with the findings for marine studies^18,19^ and thus indicate a general applicability of the described mechanism. The relation of Si and Ca availability to soil respiration has some limitations as the positive effect of Si via increasing P availability may only be important for P-limited systems. However, as Si also mobilizes organic C from soil particles^36^, making it more available for microbial decomposition, a slight increase of soil respiration by Si may also occur in non P limited systems. The negative effect of Ca on both P mobility at high soil pH^6^ and on soil respiration by stabilization of organic matter was already shown by^37,38^ to be important for soil systems in general.

**Figure 5:**
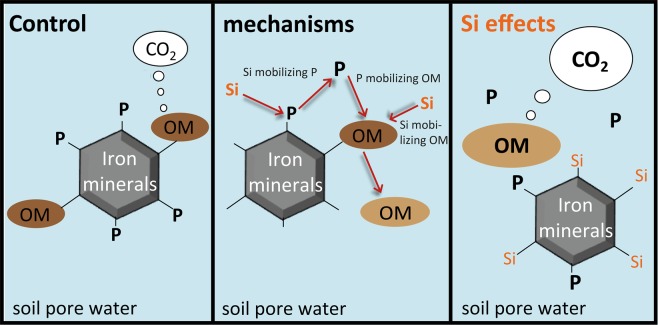
Suggested Si effects on P mobilization and soil respiration. Overview on the overall suggested effects of Si on soil P availability and potential effects on C turnover by increased P mobility and organic matter (OM) mobilization due to increased Si and P availability.

In conclusion, the significant effect of Si mobilizing inorganic P from strong binding sites, i.e. biologically unavailable fractions, highlights the importance of including Si in studies focusing on biogeochemical cycles of P. We suggest that in soils with high Si availability and high porewater Si concentration the unavailable fraction of soil inorganic P might be reduced by competition of Si and inorganic P for sorption at Fe-minerals. Furthermore, the P mobility in soils with high Si availability will be elevated as long as the effect of Si competing with inorganic P for binding sites at Fe-minerals is proceeding from the mineral surface to the core of the mineral particles. The Si effect on inorganic P sorption at the surface of Fe-minerals is fast but it may require a significant time until this process reaches the core of the mineral particles of large minerals. Hence, Si may be important for inorganic P availability in terrestrial soils in general (Fig. 5), as has already been proven for marine ecosystems. Our results improve the understanding of soil inorganic P availability and mobilization mechanisms with potential implication for C turnover (at least under P deficiency), but also by Si^36^ and P^51^ mobilizing organic C from soil particles. The described mechanism of Si mobilizing P may be particularly important for agricultural systems, where a high demand of P fertilizer exists to maintain proper plant nutrition^2^. A large portion of inorganic P in soils is strongly bound/adsorbed to Fe-oxides and Al-oxides^6^. Silicon may be able to mobilize P from these mineral surfaces, increasing P availability for plants. This may be very important for tropical soils (e.g. oxisols) as the Fe and Al content dominate soil mineralogy, leading to low P availability despite high total P content^53,54^. Agricultural systems with a high yearly Si export due to crop harvest leading to a decrease of Si availability^16,17^ may need much less P fertilizer when increasing Si availability in soils resulting in a mobilization of P from former unavailable fractions. This increase in P mobility by Si, however, may also lead to an enhanced mineralization of soil organic matter and the release of CO_2_ in P limited soils. Furthermore, the effect of Si increasing P mobilization from soil may also be an important factor controlling eutrophication of aquatic ecosystem downstream. In contrast to Si, Ca decreases P mobility by precipitating P as calcium phosphate (but only at soil pH >~7) and is reducing soil respiration and CO_2_ release due to flocculating organic matter by cation bridges. Hence, for soil in the pH range <~7 an increase of Si availability may result in increased P availability (Si effect). An additional increase in Ca availability may have no effect on P availability in this pH range but leads to a decrease in the effect of Si enhancement on CO_2_ emissions (Ca effect).

## Methods

### Soil sampling and element analysis

**Figure 6:**
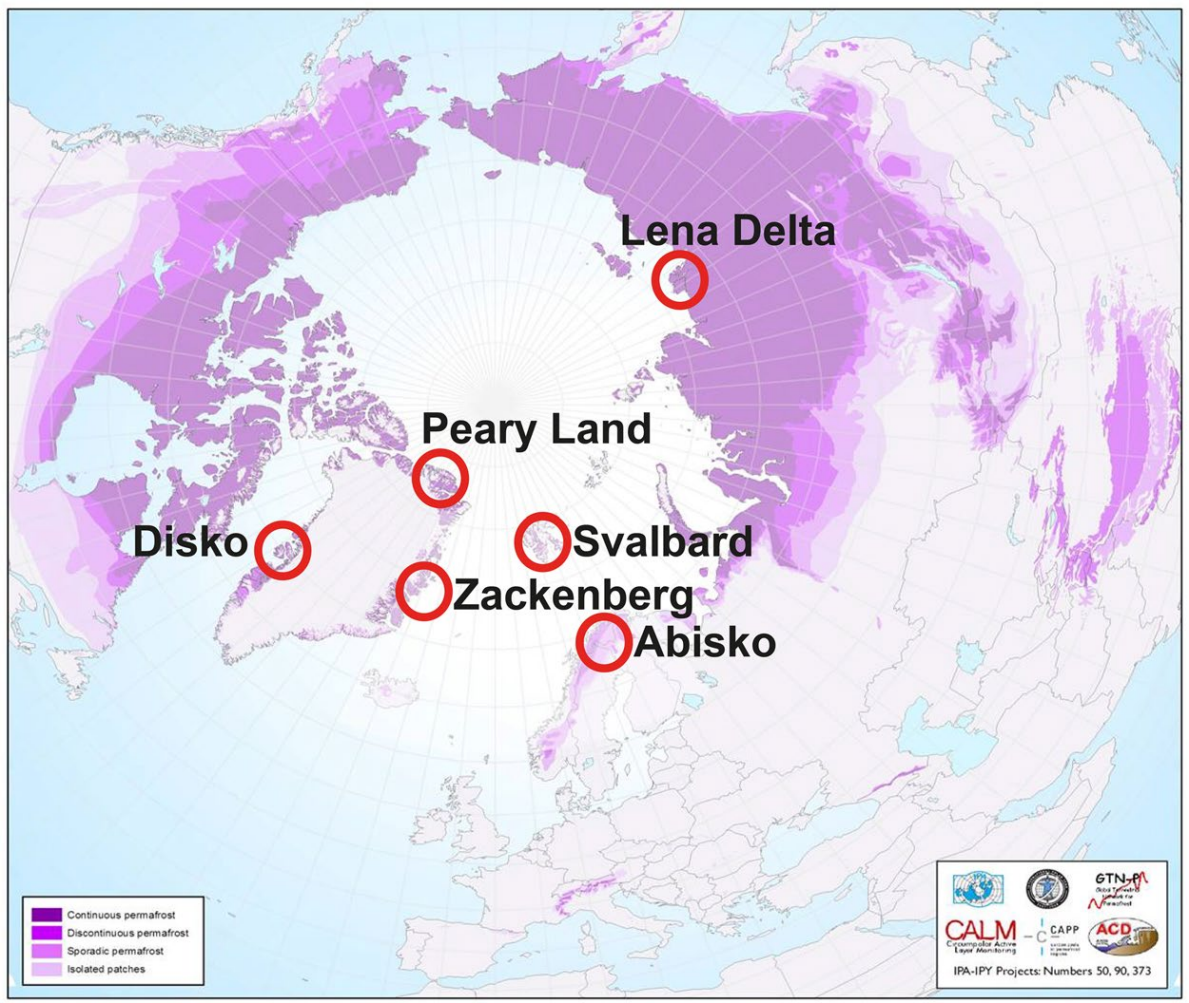
Overview on sampling locations within the Arctic. Map on distribution of permafrost soils was taken from https://ipa.arcticportal.org/publications/occasional-publications/what-is-permafrost ^64^ accessed on May 24^th^ 2018. Purple shading refers to continuous, discontinuous, sporadic to isolated permafrost (from high to lower colour intensity).

Soil samples were retrieved from the upper 1 m of soil at different locations in four Arctic regions (Lena Delta, Abisko, Svalbard, and Greenland) using an auger or spade (Fig. 6). The four Arctic regions were chosen to represent contrasting Arctic environments in terms of climate, landscape history, vegetation and expected soil organic C content/age. For the current study, we focused only on mineral soils excluding the highly organic samples from the profile surface. The soils from Lena Delta, Abisko and Svalbard were collected along 1–2 km transect that crossed all major landscape types within the respective areas. The coring locations were strictly equidistant at 100 or 200 m intervals. All samples were retrieved in frozen conditions, and subsequently transported and stored frozen. This sampling procedure was followed to avoid any subjective choice of profile location. Detailed sampling site description can be found in the supporting information.

Samples were gently crushed in their frozen state using a steel mortar. The frozen material was split and sieved in a −10 °C room through an 8 mm sieve until all particles passed through the sieve.

Measurements of Si availability was made by extraction in a 0.1 M Na_2_CO_3_ solution at 85 °C for five hours according to Struyf, *et al*.^55^ and DeMaster^56^, as this method determines the Si pool potentially cycled within the ecosystem^15^. Phosphorus and Ca availability was analysed using the Mehlich-3 extract^57^ as this is the only method for available P extraction which extracts Ca, too. Si was measured by ICP-OES, whereas P and Ca where measured by ICP-MS. All measurements were done after sample filtration (0.2 µm cellulose acetate).

### Laboratory experiments

To study the influence of Ca and Si on P availability, two representative soil samples with different Si, P, and Ca availability as well as soil pH were selected for incubation experiments. The first soil (from Peary Land) had low Si (1.4 mg g^−1^ alkaline extractable Si)^56^, high initial Ca availability (15.6 mg g^−1^ Mehlich-3 extractable Ca)^57^, and a high pH varying around ~8.4. The second soil (from Disko Island) had elevated Si (5.2 mg g^−1^), low initial Ca availability (1.7 mg g^−1^), and a pH of ~5.6. The initial P availability (Mehlich-3)^57^ was low for both soils (0.01 mg g^−1^ for Peary Land and 0.13 mg g^−1^ for Disko), whereas total P was 0.6 mg g^−1^ for Peary Land and 1.5 mg g^−1^ for Disko. Both soils (each with control) were incubated with Si addition of 0 (ambient), 3, 6, or 10 mg g^−1^ and Ca addition of 0 (ambient), 10, 20 or 30 mg g^−1^ to cover the range of soil Si and Ca availability found in the literature^14,22^. About 5 g soil were incubated in 25 mL glass vials together with 2 mL of pure water and different levels of Si as Si nano fertilizer Aerosil-300 (Evonik, Germany) and Ca (as CaO, pH adapted to solution or each soil by using HCl) at 5 °C. Two control treatments were analysed. The first was without any Si or Ca addition to check for the effect of the water addition on P mobilisation and C respiration, which in all cases was comparable (no significant differences, data not shown). The second control experiment we added chloride as NaCl to determine if the HCl added to stabilize Ca solution to initial soil pH effected P mobilisation and C respiration. The latter was also comparable (no significant differences, data not shown). To demonstrate that the positive Si effect on respiration is caused by the Si-induced increase of P availability, we conducted an additional treatment where P as NaH_2_PO_4_ was added in concentrations equivalent to the amount mobilized by the highest Si treatment. The vials were closed by Parafilm for two weeks to allow for gas exchange. Afterwards, Parafilm was replaced by butyl-rubber septa and aluminium crimp caps and purged with air. After four days the headspace gas of the vials was sampled using a 3 mL gas tight syringe with a gas tight three-way valve. These incubation experiments were run until quasi-constant soil respiration was achieved (three months) to focus on long-term effects of Si and Ca availability on P availability and potential effects on soil respiration. At the end of the experiment soil porewater samples were taken, filtered (0.2 µm cellulose acetate), and acidified using nitric acid. Si, P and Ca analysis for the porewater samples was done by ICP-MS and ICP-OES.

### CO_2_ Analysis

Gas samples were injected into a gas chromatograph (SRI Instruments 8610, SRI Germany). The sampling and analysis was repeated monthly until constant soil respiration was achieved. The CO_2_values were calculated as follows:

nCO_2_ (mol) = CO_2_ values (ppm, from instrument 10^−6^ 101325 kg m^−1^ s^−2^) * V (m^3^) (R (8.3144 kg m^2^ s^−2^ mol^−1^ K^−1^)^−1^ (T (K))^−1^. After three months, a constant CO_2_ release was observed (same values as one month before, data not shown).

### X-ray spectromicroscopy

To identify the chemical mechanism of the observed P-release by Si, spatially resolved speciation analysis was done for the Peary Land soil after three months of incubation (laboratory experiments) using soft X-ray spectromicroscopy. As sorption processes were likely to be involved in P binding, we used a transmission approach that allows for the quantitative specific mapping of Fe-bearing phases with sub-particle spatial resolution. We used synchrotron-based scanning transmission X-ray microscopy (STXM), a spatially resolved spectromicroscopy approach that combines near edge X-ray absorption fine structure (NEXAFS) spectroscopy with a spatial resolution of tens of nm. We selected this approach because we expected a “surface”-related sorption or complexation process in the uppermost tens of nanometers to be responsible for the Si-binding / P-release. This range is difficult to target with bulks spectroscopic approaches such as bulk NEXAFS spectroscopy where the high concentrations of the respective chemical species that are not changed by the process, would completely cover potential spectral changes that originate solely from close to the surface. P1s spectroscopy with fluorescence detection for example would probe the uppermost micrometers of the sample and thus it would very unlikely be able to pick up the expected subtle spectral changes at the P1s absorption edge and it would not be possible get valuable synchrotron-beamtime allocated for a project that is unlikely to obtain results. Spatially resolved NEXAFS spectroscopy in an energy range of soft X-rays that are readily absorbed by tens of nm of material, however, appeared to be most promising according to our absorbance simulations with aXis2000^58^ based on the atomic scattering factors^59^. Therefore, we considered absorption edges between 200–1000 eV of elements that are likely involved in the process and decided on the Fe2p absorption edges at 706.8 and 719.9 eV as Fe-minerals are the most likely binding sites for inorganic P in soils. STXM allows to derive local NEXAFS spectra from edges of individual particles where it would probe the previously mentioned uppermost tens of nm at the required sensitivity. Soil particles were suspended in deionized water and wet-deposited onto Formvar coated, 300 mesh Cu TEM grids (Plano GmbH, Wetzlar, Germany) and instantly dried. Randomly selected areas on the grid were analysed using the STXM at beamline 10ID-1 of the Canadian Light Source^60^. Image-stacks were recorded across the Fe 2p absorption edges from 699 eV to 740 eV with an energy resolution of 0.18 eV in the energy region of interest. The resulting datasets were analysed using aXis2000^58^. The stacks were aligned and converted from transmission to linear absorbance using the following formula:

$${\rm{OD}}=-\,\mathrm{ln}({\rm{I}}/{{\rm{I}}}_{0})$$

where OD is the optical density, I is the intensity at a specific pixel, I_0_ is the intensity of the X-ray beam in an empty region adjacent to the sample. The resulting image stack was averaged across the entire energy range to obtain the best quality image in the region of interest. Masks were then extracted from the average image based on the following OD-ranges: 0.01–0.1, 0.1–0.3, 0.4–0.6, 0.7–0.9, 1.0–1.2 (Fig. 3A–C); average spectra for these OD-ranges were extracted from the image stacks using these masks (examples in Fig. 3D for the original soil (dark colours) and after Si addition (bright colours)). Thicker regions were omitted from the analysis to avoid potential problems with absorption saturation that may affect the quality (non-linearity) of the extracted spectra^61^.

The resulting spectra were analyzed by linear combination fitting using various combinations of two spectra of known reference compounds and a sloped background representing non-specific absorption of non-Fe elements at the Fe2p edge. The following phases were used: Fe(III)-minerals ferrihydrite, goethite, Fe(II)-minerals siderite and vivianite (Fig. 3E)^62,63^. The lowest standard deviations of the individual fits were obtained by a combination of the spectra of ferrihydrite and vivianite. The spectra were previously normalized to a 1 nm layer of the respective compound to obtain quantitative maps representing cumulative thickness [nm] of the respective compounds. Two independent datasets of each sample type were analysed, shown in Fig. 3. For each dataset and each thickness range, based on the masks, the fraction of an Fe(II)-phase and the total Fe were calculated and plotted against the cumulative thickness of total Fe (Fig. 3F). Here, we used a mixture of exactly one Fe(II) and one Fe(III) phase to minimize the standard deviation of the fits (i.e. vivianite and ferrihydrite).

## Supplementary information

Supplementary Dataset 1

## Data Availability

All data analyzed during this study are included in this published article.
