# Supplementary material for: Silicon increases the phosphorus availability of Arctic soils
Source: Sci Rep. 2019 Jan 24;9:449. doi: 10.1038/s41598-018-37104-6 (PMC6345794; doi:10.1038/s41598-018-37104-6)
Supplement: Supplementary file 1 — Supplementary Dataset 1 [file 41598_2018_37104_MOESM1_ESM.doc]

Supplementary Material for

Silicon increases phosphorus availability of Arctic soils

Jörg Schaller^1*^, Samuel Faucherre^2^, Hanna Joss^1^, Martin Obst^3^, Mathias Goeckede^4^,Britta Planer-Friedrich^1^, Stefan Peiffer^5^, Benjamin Gilfedder^6^, and Bo Elberling^2^

^1^Environmental Geochemistry, Bayreuth Center for Ecology and Environmental Research (BayCEER), University Bayreuth, 95440 Bayreuth, Germany

^2^Center for Permafrost (CENPERM), University of Copenhagen, DK-1350 Copenhagen, Denmark

^3^Experimental Biogeochemistry, BayCEER, University Bayreuth, Dr.-Hans-Frisch-Str. 1-3, 95448 Bayreuth

^4^Max Planck Institute for Biogeochemistry, Hans-Knöll-Straße 10, 07745 Jena, Germany

^5^ Department of Hydrology, BayCEER, University of Bayreuth, D-95440 Bayreuth, Germany

^6^Limnological Research Station, Bayreuth Center for Ecology and Environmental Research (BayCEER), University Bayreuth, 95440 Bayreuth, Germany

Jörg Schaller

Email: Joerg.Schaller@uni-bayreuth.de

Supplementary Material Text

**Sampling site description**

Svalbard (Adventdalen valley and the surroundings of Ny-Ålesund), was chosen due to low soil organic C content representing a wide area of the Arctic. The area was glaciated during the last glaciation ^1^ and consequently the soil organic C is younger than non-glaciated areas of the Arctic. The Lena Delta (First and Third terrace) was chosen to represent mid to high soil organic C content representing several inland Arctic areas known as Yedoma. These areas were not glaciated during the last Ice Age and extend over large plateaus in Siberia ^2^. Thus, the soil organic C is the oldest of the Arctic; the organic matter in soils was deposited continuously due to the absence of glaciation. Abisko (Stordalen Palsa mire) was chosen to represent a high soil organic C content environment representing a smaller part of the Arctic. The Abisko site is characterised by the presence of large peatland areas in discontinuous permafrost ^3^. For more detailed information about the soils and temperature from Lena Delta, Abisko and Svalbard see Faucherre, et al^. 4^.

The soil at three different sites from Greenland (Jørgen Brønlund Fjord in North Greenland, Zackenberg in Northeast Greenland and Disko in West Greenland) were collected from the uppermost mineral soil layers. Jørgen Brønlund Fjord is located on Peary Land in North Greenland about 700 km south of the North Pole (82°07'N; 33°04'W) and the most northerly ice-free region in the world. The area is dominated by bedrock formed during Proterozoic to Ordovician and made of coarse sandstone and carbonates. In low lands and on slopes with running melt water from snowdrift, a thin soil layer up to 20 cm has developed, but is mostly less than 5 cm deep. The site is classified as mountainous polar desert with a growing season generally covering only about one month (July) and an annual precipitation less than 100 mm per year ^5^. The mean annual air temperature in the central part of the fiord is -13.6 °C (2014-2016).

Zackenberg is located in the Zackenberg heath plain lowlands (74°30'N / 20°30'W) in High Arctic Northeast Greenland. The valley is flat and dominated by non-calcareous sandy fluvial sediment and Aeolian sand. The area lies within the zone of continuous permafrost with a mean annual air temperature around -10 °C and annual precipitation of around 150 mm. Given the low summer precipitation, water availability during the growing season is mainly controlled by snow-melt from large snow-patches. Dense vegetation patches and seasonally water-logged grasslands are formed below the snow patches, whereas the remaining parts of the landscape are relatively dry throughout the growing season. Soil temperatures at a depth of 5 cm are below -18 °C for about four months and above 0 °C for about 120 days per year. Maximum annual thaw depths on the moist tundra heath and are approximately 70 cm deep with a progressive permafrost degradation and vertical active layer extension of about 0.9 cm yr-1 in the period 1997-2007. Additional information can be found in ^6^.

The Disko site in West Greenland is in the Blæsedalen valley (69°16’N, 53° 27’W) with a mean annual soil temperature at 5 cm depth of -0.9 °C (1991-2004) ^7,8^. The warmest monthly mean air temperature is 7.9 ± 1.6 °C (SD) during July, whereas the coldest monthly mean is -14.0 ± 5.0 °C during February-March (Hollesen et al., 2015). Frozen soil conditions prevail from October to late May. The area is characterized by presence of discontinuous permafrost and the soil material is <10,000 years old. Soil development is generally weak and dominated by the basaltic rocks in the area ^9^.

**Table S1** Statistical data (ANOVA) of Si and Ca effects on both P mobilization and soil respiration. Effects of Si and Ca on P mobilization into soil porewaters and soil respiration for the soils from Peary Land and Disko. Degree of freedom (df) and F is referring to the F value which assess the equality of variances.

| **P mobilization** |  |  |  |  |  | **Soil respiration** |  |  |  |  |
| --- | --- | --- | --- | --- | --- | --- | --- | --- | --- | --- |
| **Peary Land** |  | **df** | **F** | ***P*** |  | **Peary Land** |  | **df** | **F** | ***p*** |
|  | **Si** | 3 | 252.024 | **<0.001** |  |  | **Si** | 3 | 12.896 | **<0.001** |
|  | **Ca** | 3 | 156.761 | **<0.001** |  |  | **Ca** | 3 | 107.201 | **<0.001** |
|  | **Si x Ca** | 9 | 28.996 | **<0.001** |  |  | **Si x Ca** | 9 | 3.798 | **<0.001** |
|  |  |  |  |  |  |  |  |  |  |  |
|  |  |  |  |  |  |  |  |  |  |  |
| **Disko** |  |  |  |  |  | **Disko** |  |  |  |  |
|  | **Si** | 3 | 448.095 | **<0.001** |  |  | **Si** | 3 | 3.393 | **0.026** |
|  | **Ca** | 3 | 41.076 | **<0.001** |  |  | **Ca** | 3 | 35.842 | **<0.001** |
|  | **Si x Ca** | 9 | 6.059 | **<0.001** |  |  | **Si x Ca** | 9 | 2.565 | **0.018** |

**References**

1. Landvik, J. Y. *et al.* The last glacial maximum of Svalbard and the Barents Sea area: ice sheet extent and configuration. *Quaternary Science Reviews* **17**, 43-75 (1998).

2. Schwamborn, G., Rachold, V. & Grigoriev, M. N. Late Quaternary sedimentation history of the Lena Delta. *Quaternary international* **89**, 119-134 (2002).

3. Åkerman, H. J. & Johansson, M. Thawing permafrost and thicker active layers in sub‐arctic Sweden. *Permafrost and Periglacial Processes* **19**, 279-292 (2008).

4. Faucherre, S. *et al.* Short and long-term controls on active layer and permafrost carbon turnover across the Arctic. *Biogeosciences* (2018).

5. Bennike, O. *Quaternary geology and biology of the Jørgen Brønlund Fjord area, North Greenland*. Vol. 18 6-15 (Kommissionen for Videnskabelige Undersøgelser i Grønland, 1987).

6. Jørgensen, C. J., Johansen, K. M. L., Westergaard-Nielsen, A. & Elberling, B. Net regional methane sink in High Arctic soils of northeast Greenland. *Nat. Geosci.* **8**, 20 (2015).

7. Hansen, B. U., Elberling, B., Humlum, O. & Nielsen, N. Meteorological trends (1991–2004) at Arctic Station, Central West Greenland (69 15'N) in a 130 years perspective. *Geografisk Tidsskrift-Danish Journal of Geography* **106**, 45-55 (2006).

8. Hollesen, J. *et al.* Winter warming as an important co‐driver for Betula nana growth in western Greenland during the past century. *Glob. Change Biol.* **21**, 2410-2423 (2015).

9. D'Imperio, L., Nielsen, C. S., Westergaard‐Nielsen, A., Michelsen, A. & Elberling, B. Methane oxidation in contrasting soil types: responses to experimental warming with implication for landscape‐integrated CH_4_ budget. *Glob. Change Biol.* **23**, 966-976 (2017).
